# Structural Elucidation of Agrochemicals and Related Derivatives Using Infrared Ion Spectroscopy

**DOI:** 10.1021/acs.est.2c03210

**Published:** 2022-10-10

**Authors:** Matthias J.A. Vink, Fred A.M.G. van Geenen, Giel Berden, Timothy J. C. O’Riordan, Peter W.A. Howe, Jos Oomens, Simon J. Perry, Jonathan Martens

**Affiliations:** † FELIX Laboratory, Institute for Molecules and Materials, Radboud University, Toernooiveld 7, 6525ED Nijmegen, the Netherlands; ‡ Syngenta, Jealott’s Hill International Research Centre, RG42 6EY, Bracknell, Berkshire, United Kingdom

**Keywords:** infrared ion spectroscopy, mass spectrometry, azoxystrobin, benzovindiflupyr, agrochemicals, transformation products

## Abstract

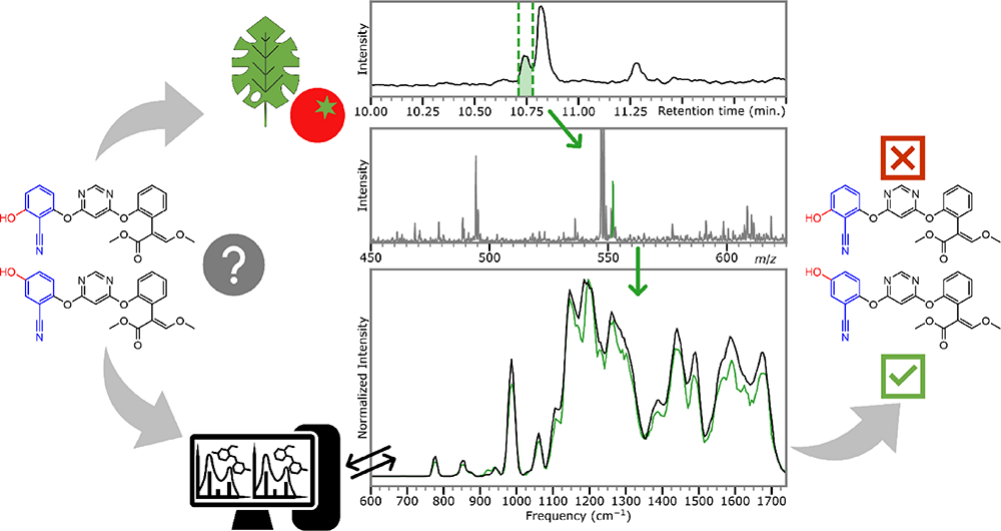

Agrochemicals frequently undergo various chemical and metabolic transformation reactions in the environment that often result in a wide range of derivates that must be comprehensively characterized to understand their toxicity profiles and their persistence and outcome in the environment. In the development phase, this typically involves a major effort in qualitatively identifying the correct chemical isomer(s) of these derivatives from the many isomers that could potentially be formed. Liquid chromatography-mass spectrometry and nuclear magnetic resonance (NMR) spectroscopy are often used in attempts to characterize such environment transformation products. However, challenges in confidently correlating chemical structures to detected compounds in mass spectrometry data and sensitivity/selectivity limitations of NMR frequently lead to bottlenecks in identification. In this study, we use an alternative approach, infrared ion spectroscopy, to demonstrate the identification of hydroxylated derivatives of two plant protection compounds (azoxystrobin and benzovindiflupyr) contained at low levels in tomato and spinach matrices. Infrared ion spectroscopy is an orthogonal tandem mass spectrometry technique that combines the sensitivity and selectivity of mass spectrometry with structural information obtained by infrared spectroscopy. Furthermore, IR spectra can be computationally predicted for candidate molecular structures, enabling the tentative identification of agrochemical derivatives and other unknowns in the environment without using physical reference standards.

## Abstract 2

Characterizing agrochemical transformation products and related compounds remains a major challenge using currently applied analytical methodologies. We demonstrate a new approach using infrared ion spectroscopy to identify the molecular structures of isomeric compounds and unknowns.

## Introduction

Agrochemicals are used to protect crops from adverse effects and are usually applied in formulations onto soil and crops.^1−4^ However, chemical derivatives often arise following their application, produced by the crop itself, by the physical environment, or by pests and microorganisms. The complete profile of these derivatives must be established in order to assess their impact on environmental and human safety and to support the registration of new agrochemicals.^1,3−7^ However, these transformation reactions can frequently result in several possible chemical isomers that must be distinguished. Thus, at the research and development phase, a major challenge is often the qualitative identification of agrochemical derivatives to be able to provide detailed regio- and stereochemical information crucially used to guide the synthesis of (internal) reference standards that are required to develop validated quantitative methods for, among other purposes, residue analysis.

From an analytical perspective, this involves analyzing low-level (often micromolar or lower) compounds from complex biological/environmental sample matrices such as plants, human or animal body fluids/tissues, soil, and water samples.^8−11^ Liquid chromatography (LC) and gas chromatography (GC) are commonly used to separate complex mixtures before analysis, typically by mass spectrometry (MS).^12−17^ This approach works well for detecting and quantifying known compounds but faces many potential challenges when identifying unknowns and distinguishing closely related isomers. Tandem mass spectrometry (MS/MS) is commonly used to provide additional certainty in identifying molecular structures; fragmentation mass spectra of unassigned features of interest can be matched to MS/MS databases.^18−20^ However, closely related isomeric compounds often have similar fragmentation spectra and retention times. Another challenge in this approach is that MS/MS fragmentation pathways remain challenging to predict in silico, and MS/MS spectra databases must be constructed based on measurements of reference standards.^21,22^ Thus, identifying unknown compounds where standards are not commercially available often involves lengthy and costly syntheses of several candidate compounds to confirm a structural assignment.

Nuclear magnetic resonance (NMR) spectroscopy can be used as an alternative technique to elucidate molecular structures, but its limited sensitivity and selectivity compared to MS restrict its applicability to compounds available in microgram or greater amounts in relatively pure form. This often leads to time-consuming purification and concentration procedures, making its use for the analysis of complex biological samples less appealing. Infrared (IR) spectroscopy can often differentiate structural isomers based on the unique vibrational frequencies that constitute a so-called infrared fingerprint. Nevertheless, IR spectroscopy cannot distinguish between the signals arising from multiple compounds in complex mixtures. However, when combined with MS, one can take advantage of the selectivity of (LC-)MS, isolating the individual ion population (*m*/*z*) of interest from all other species present in the sample and matrix before measuring the IR spectrum. This selectivity and sensitivity make it possible to characterize compounds at the relatively low abundances (<5 μg/mL) typically found in qualitative identification studies and typically requires less than 10 μL of sample for analysis. This technique, referred to as IR ion spectroscopy (IRIS), can be implemented on various MS platforms using IR multiple-photon dissociation (IRMPD), enabling one to record the IR fingerprint of a mass-selected ion population. This is commonly done using a quadrupole ion trap (QIT) and intense and tunable IR lasers, such as a free-electron laser at a large-scale user facility or a compact optical parametric oscillator (OPO) source.^23−29^

Here, we assess the viability of using IRIS to differentiate and identify two agrochemicals (azoxystrobin and benzovindiflupyr) and several of their hydroxylated derivatives by comparing their IR ion spectra with computationally predicted spectra. We demonstrate the characterization of the compounds from spiked tomato and spinach quality control matrices and envision that the method can analogously be extended to other environmental and biological samples.

## Chemicals and Materials

LC–MS-grade acetonitrile (MeCN), water, and formic acid (FA) were obtained from Merck (Darmstadt, Germany). Benzovindiflupyr (≥99.5%) was obtained from LGC Standards (Augsburg, Germany), and azoxystrobin (≥95.8%) was synthesized by Syngenta (Bracknell, UK). Hydroxylated derivatives of benzovindiflupyr (SYN546039, SYN546040, and SYN546060, in this study, referred to as BvOH-1, BvOH-2, and BvOH-3, respectively) were synthesized at Syngenta and characterized to have 98, 97, and 98% purity levels, respectively. Hydroxylated azoxystrobin derivatives (SYN550684, R400299, and R400297, in this study, referred to as AzOH-1, AzOH-2, and AzOH-3, respectively) were synthesized at Syngenta and characterized to have 90, 97, and 96% purity levels, respectively. Stock solutions of all compounds were made at the millimolar level in MeCN/H_2_O (50/50 v/v%) and kept at −20 °C for storage. All samples were prepared from these stock solutions by thawing to room temperature and diluting further in MeCN where required. For the Cs^+^ adducts, 1 μL of 86 mM CsCl was added to the solution before infusion.

### Preparation of Spiked Matrices

All quality control matrices were provided by Syngenta (Bracknell, UK) and were prepared from untreated crop samples. A spinach leaf matrix was prepared from liquid-nitrogen frozen spinach leaves, which were ground to a powder using a mortar and pestle, after which 0.5 g of crude extract was added to 2.0 mL of MeCN and agitated by hand. Subsequently, the solution was centrifuged at 10,000 rpm for 10 min in Eppendorf vials. A decant supernatant was transferred to HPLC vials with a final solvent composition of 1:4 H_2_O:MeCN, where the water content originates from the leaf material. Tomato matrix samples were prepared by dividing four trays of macerated red tomatoes into approximately 300 samples stored at −80 °C until the preparation of the final matrix. Crude extract (2.5 mL) was thawed to room temperature, after which it was diluted 1:1 with MeCN and agitated by hand. Subsequently, it was centrifuged at 10,000 rpm for 10 min in Eppendorf vials. The decant supernatant was transferred to HPLC vials. Both matrix samples were stored at −20 °C until required, upon which they were thawed to room temperature. Subsequently, 250 μL of matrix sample was spiked with 1 to 8% volume of the relevant agrochemical stock solution for analysis, minimizing the matrix’s dilution. In Figures SI 7 and SI 8, we show that no isobaric interferences are present in the quality control matrices in the elution range of the hydroxylated derivatives.

## Methods

### (LC)-MS

A Bruker Elute HPLC system was used with a column oven and autosampler for separation and fractionation. The autosampler was held at 4 °C, and the column oven was held at 40 °C during separation on a Waters ACQUITY UPLC HSS T3 reversed-phase C18 column with dimensions of 2.1 × 150 mm with 1.8 μm particles with a 100 Å pore size on which injections of 2 μL were made. Elution was performed under a linear gradient from 95% solvent A (0.1% FA in water) and 5% solvent B (0.1% FA in MeCN) at a flow rate of 0.4 mL/min to 5% solvent A and 95% solvent B in 15 min. These conditions were held for an additional 5 min before being switched back to the initial conditions in 1 min and kept for an additional 5.5 min to allow for equilibration of the column. The LC was coupled to a Bruker amaZon QIT, equipped with a two-position six-port divert valve. To fraction the agrochemical derivative of interest from the biological matrix, the elution time was first confirmed using the QIT. Subsequently, five injections were fractioned by programming the divert valve to divert the flow to a sample vial at the observed elution time. The acquired fractionated sample was diluted approximately 1:1 by volume in MeCN before direct infusion ESI using a Hamilton 250 μL syringe.^30,31^

### IRIS Measurements

To perform the IRIS experiments, the FELIX IR-FEL was set to scan over a frequency range of 550–1850 cm^–1^ in steps of 3 or 5 cm^–1^. A modified Bruker amaZon QIT was used, which allows for optical access to the trapped ion population by the IR radiation of FELIX. The QIT was modified to synchronize to the FELIX IR-FEL to perform IRIS measurements, which have previously been described in detail.^32^ The IRIS spectra were measured from mass isolated ions irradiated with a single IR laser pulse from which the photo-dissociation yield is calculated, averaging four to eight fragmentation spectra.^32^

An IRIS spectrum is constructed from a series of mass spectra by monitoring the characteristic fragments as the IR frequency is tuned. When the frequency of the IR laser is resonant with an absorption band of the trapped ions, photodissociation occurs, and fragment ions are detected in the mass spectrum. The IR dissociation yield can be calculated from the ion intensity of the precursor (*I*_p_) and fragment ions (∑*I*_F_) after irradiation by taking the natural logarithm. The yield as defined in eq 1 is directly proportional to the ions’ dissociation rate and can thus be interpreted more closely as the vibrational absorption spectrum of the ions.^33^ An experimental IR spectrum is obtained by plotting the yield as a function of IR frequency (eq 1). The wavelength was calibrated using a grating spectrometer, and the yield was linearly corrected for frequency-dependent variations in laser pulse energy.
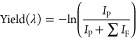
1

This experimental IR spectrum can then be matched to reference IR spectra measured from physical standards or obtained from computational predictions generated using quantum chemistry. IRIS spectra are, in most cases, similar to linear absorption IR spectra that are calculated by quantum-chemical software after an appropriate line shape convolution.^25−27,29,30,34−37^ Typically, predicted IR spectra at the density functional theory (DFT) level are sufficiently accurate to allow for preliminary structural assignments without the use of physical standards. While the comparison to the IR spectra measured for physical reference standards often remains desirable for definitive identification of the molecular structures of unknowns, a tentative assignment that narrows down the range of candidate isomers can significantly reduce the required synthetic efforts when physical standards are unavailable.^30,35,37−43^

### Quantum-Chemical Computation of IR Spectra

A conformational-search workflow was used to determine the lowest-energy geometries for the molecular structures of the agrochemicals and their derivatives.^30,44^ This workflow uses the cheminformatics toolbox RDKit in Python 3,^45^ in which all oxygen and nitrogen atoms were considered protonation or deprotonation sites or to coordinate with a Cs^+^ ion. Input structures formulated as SMILES codes are converted to 3D conformers; a distance geometry algorithm was employed to probe the conformational space for 500 random 3D conformations, which were then minimized by employing the MMFF94 force field. Subsequently, these conformations were further optimized at the semi-empirical PM6 level, followed by vibrational analysis. The generated PM6-optimized conformers were filtered for duplicates, and structures with broken bonds were omitted. In addition, a relative energy cutoff of 40 kJ/mol was used, based on the Gibbs free energies found using PM6, filtering out unfavorable structures. Further, we set a limit of 20 conformers per structure for further calculations at the DFT level. The selected conformers of each structure were optimized using the B3LYP/6–31++G(d,p) level of theory, improving the accuracy of the computed geometries and vibrational frequencies compared to PM6. To better predict the electronic energy, a Møller–Plesset second-order correction was calculated, substituting those found using B3LYP except for the Cs^+^ adducts for which the B3LYP results were used. For the Cs^+^ adducts, an MWB46 effective core potential was used to compliment the 6–31++G(d,p) basis set for the Cs atom. All calculations were carried out using the Gaussian 16 suite of programs.^46^

The IR frequencies were scaled using a 0.975 factor. Calculated line spectra were broadened by a Gaussian function of 20 cm^–1^ full width at half maximum to help compare the obtained theoretical spectra with those found experimentally. DFT-computed IR spectra for all structures of each isomer were compared to measured IR spectra. Based on the degree of IR spectral match and relative energy, we assigned a single calculated structure. In some cases, it may be that more than one low-energy structure of a given isomer contributes to the measured IR spectrum of the mass-isolated ion population; however, here, we focus on the differentiation of different isomers rather than the assignment of specific conformations. We have normalized all spectra to facilitate comparing experimental spectra and their computed counterparts.

## Results and Discussion

### Benzovindiflupyr Hydroxylated Products

**Figure 1:**
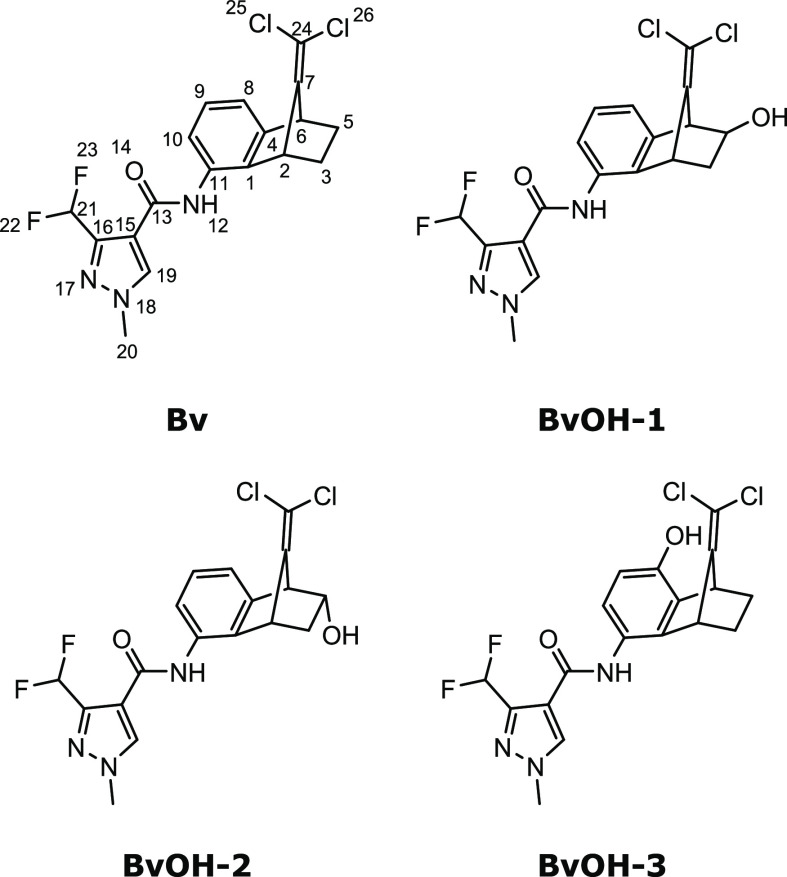
Chemical structures of benzovindiflupyr and related oxidized structures.

Figure 1 depicts benzovindiflupyr (**Bv**) and three hydroxylated derivatives. **BvOH-1** and **BvOH-2** are diastereomers resulting from oxidation at C-5, whereas **BvOH-3** is a structural isomer where the oxidation site is located on the aromatic ring in the para position relative to the amide group. While the three hydroxylated derivatives have the same *m*/*z*, the **BvOH-3** compound has a phenolic hydroxyl group with a lower pKa value than the cyclohexanol of the other derivatives. This makes it likely that a difference in protonation and deprotonation sites exists for the two oxidation locations, which is expected to result in large differences in their IR spectra. We note that the MS/MS fragmentation spectrum of **BvOH-3** is known to differ from the other derivatives (Figure SI 5); however, differentiation of **BvOH-1** and **BvOH-2** is more challenging based on their MS/MS spectra.

**Figure 2:**
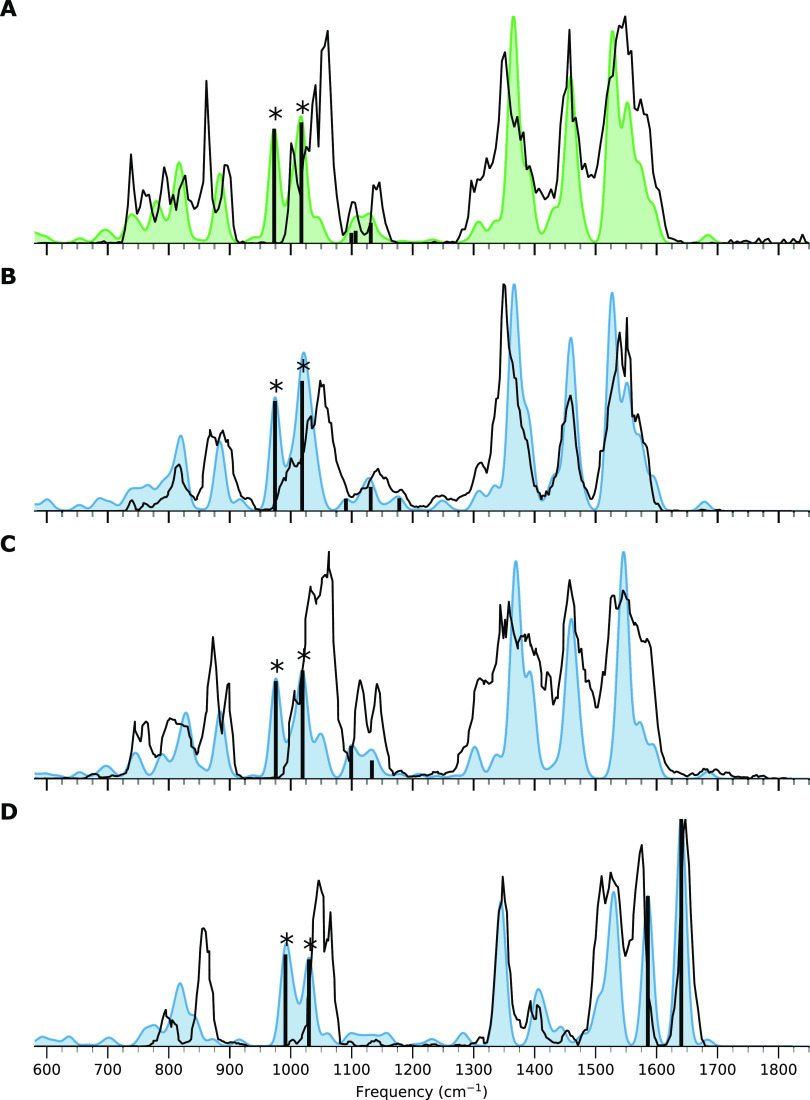
IRIS spectra of deprotonated benzovindiflupyr and oxidized derivatives. Computed absorption lines indicated by ‘*’ are vibrations related to CF_2_, which are not well predicted by the used B3LYP as described in the text; all remaining absorption lines relate to vibrations discussed in the text. **A**: IRIS spectrum of **Bv** (black trace) and predicted IRIS spectrum (green filled curve), **B**: IRIS spectrum of **BvOH-1** (black trace) and predicted IRIS spectrum (blue-filled curve), **C**: IRIS spectrum of **BvOH-2** (black trace) and predicted IRIS spectrum (blue filled curve), and **D**: IRIS spectrum of **BvOH-3** (black trace) and predicted IRIS spectrum (blue-filled curve).

The IR ion spectra of all four compounds were recorded in both positive and negative ion modes, where the negative ion mode proved to distinguish the four compounds better and is therefore discussed below. The recorded IR spectra of the deprotonated ions are depicted as black traces in Figure 2, where the matched computed spectrum for each compound is shown as a colored spectrum in the same panel. Vertical lines indicate a selection of computed vibrational bands discussed in further detail in the text below.

We observe in Figure 2 that the spectrum of the **BvOH-3** deprotonated ion, isolated at 412 *m*/*z*, is significantly different from the other three spectra. As suggested above, examination of the structure of the assigned computed spectrum confirms that **BvOH-3** deprotonates on the phenol, whereas the other compounds deprotonate on the amide group. This significantly impacts the molecule’s vibrational modes, which is reflected by the features of the IRIS spectrum predominantly in the region between 1500 and 1700 cm^–1^. Examination of the computed vibrations in this region shows that these vibrations correlate directly with the amide group’s C=O stretch at 1640 cm^–1^, which is only observed for the non-deprotonated amide. Further, a benzene CC stretch mode of the deprotonated phenol at 1590 cm^–1^ is unique compared to the other derivatives.

The IR spectra for deprotonated **BvOH-1** and **BvOH-2**, both isolated at *m*/*z* 412, show more subtle differences but are distinct. For instance, the peak structure observed between 1100 and 1200 cm^–1^ can differentiate the two isomers. In the **BvOH-1** derivative, a triplet of peaks is observed, while only two peaks are seen in the IR spectra of **BvOH-2** and **Bv**. The first is the CH_2_ twisting of a carbon of the bicyclo[2.2.1]heptane moiety in combination with the OH bending of the oxidation site and some minor delocalized CH bending vibrations. The second vibration is an in-plane CH bending vibration within the pyrazole moiety combined with a CH bending vibration of the CF_2_ group. The third vibration, only observed for **BvOH-1** in this region, is attributed to a second CH bending mode on the bicyclo[2.2.1]heptane ring system, which is not present in the spectrum of **BvOH-2**.

Interestingly, while the computationally predicted spectra in Figure 2 generally reproduce the experiments well, they show a peak shift for the doublet observed around 1000 cm^–1^ for all compounds. The mismatch is attributed to an incorrect scaling factor for the specific symmetric and antisymmetric stretching of the CF_2_ group in the molecules, but as all four compounds share this group, these vibrations are less critical in the characterization.

Lastly, note that due to the chirality of the bicyclo[2.2.1]heptane ring system prior to oxidation, as can be derived from the structure of Figure 1, two enantiomers of each oxidized product may be produced that would require chiral separation. The samples used here were synthesized as racemic mixtures, described in the Supplementary Information. However, in this study, we are only considering the relative configuration of the oxidation site.

### Azoxystrobin Hydroxylated Products

**Figure 3:**
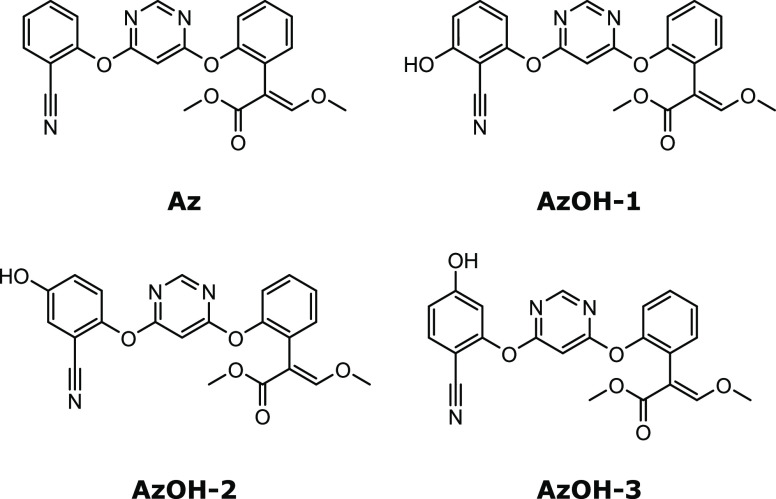
Chemical structures of azoxystrobin and related oxidized structures.

Figure 3 depicts azoxystrobin (**Az**) and its three hydroxylated derivatives: **AzOH-1**, **AzOH-2**, and **AzOH-3**. The oxidation sites are located on the *ortho*-, *meta*- and *para*- positions relative to the nitrile group, and the three isomers are not easily chromatographically separated or differentiated based on their MS/MS spectra (Figure SI 6). Thus, these three hydroxylated compounds serve as an example of closely related structures that need to be analytically differentiated but are challenging for LC–MS(/MS) approaches.

**Figure 4:**
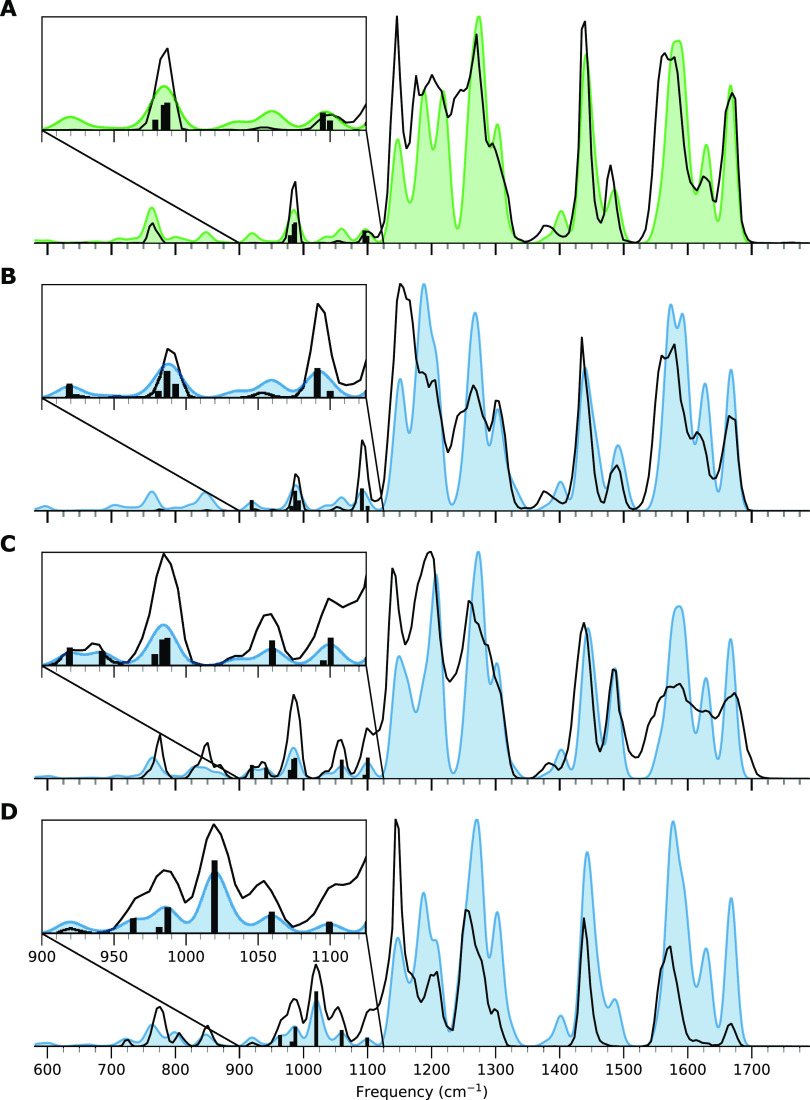
IRIS spectra of azoxystrobin and oxidized derivatives as [M + Cs]^+^ adducts. Computed absorption lines relate to vibrations discussed in the text. All panels have a subpanel depicting the wavenumber range between 900 and 1125 cm^–1^**A**: IRIS spectrum of azoxystrobin **Az** (black trace) and predicted IRIS spectrum (green-filled curve), **B**: IRIS spectrum of **AzOH-1** (black trace), and predicted IRIS spectrum (blue-filled curve), **C**: IRIS spectrum of **AzOH-2** (black trace) and predicted IRIS spectrum (blue-filled curve), **D**: IRIS spectrum of **AzOH-3** (black trace) and predicted IRIS spectrum (blue-filled curve).

Each compound was directly infused into the QIT, and the IR spectra of the protonated, deprotonated, and Cs^+^ adducts were recorded. In this case, while distinguishable, the IR ion spectra of the protonated and deprotonated ions did not show significant differences between the compounds. The IR ion spectra of the Cs^+^ adducts, isolated at *m*/*z* 552, are depicted in Figure 4 as black traces and have more significant differences, discussed in further detail below. Figure 4 shows the computed spectra as colored spectra, and vertical lines are shown for selected computed vibrational bands discussed in more detail.

The Cs^+^ adducts were studied in this case because we have previously demonstrated that this is a way of enhancing spectral differences between *ortho*-, *meta*-, and *para*-isomers.^38^ Furthermore, the [M + Cs]^+^ adducts tend to give ‘cleaner’ spectra as the Cs ion binds less strongly than other metal adducts, such as sodium, and acts somewhat like a ‘messenger tag.’^47−50^ Lastly, the relatively high mass of the Cs^+^ IRMPD product ions can easily be detected above the low mass cutoff inherent to QIT.

From Figure 4, it can be observed that a peak at 990 cm^–1^ is observed in all four spectra. This peak can be attributed to CH bending vibrations of the pyrimidine and one CO ether stretching vibration of the methyl (E)-3-methoxy acrylate group, along with some minor delocalized vibrations. All derivatives have more features in the region between 900 and 1125 cm^–1^, but this is the only peak with significant intensity for **Az**.

The **AzOH-1** and **AzOH-2** derivatives’ calculated spectra are more similar but have a few fingerprint features to differentiate the two compounds. In the **AzOH-2** spectrum, a weak doublet feature is observed at 915 and 945 cm^–1^. The first band is due to a CO ester stretch of the methyl (*E*)-3-methoxy acrylate group present in both derivatives, and the latter is an out-of-plane CH bending vibration on the oxidized benzene moiety only predicted in the **AzOH-2** derivative. However, when the experimental IR spectra of the reference standards are compared, these features are observed at relatively low intensities. Nevertheless, more significant differences between the two compounds can be observed than expected from the predicted spectra. In **AzOH-2,** the in-plane CH bending vibration of the methyl (*E*)-3-methoxy acrylate-substituted benzene at 1050 cm^–1^ is significantly more intense, whereas, in **AzOH-1,** it is almost unobserved. Further, we can look at the peak shapes between 1090 and 1225 cm^–1^, where the first peak at 1090 cm^–1^ is present in all the azoxystrobin molecules. This peak is attributed to in-plane CH bending on the benzene moieties. However, for the **AzOH-2** and **AzOH-3** derivatives, it is attached to the doublet of peaks of 1150 and 1200 cm^–1^, where it is baseline separated in **Az** and **AzOH-1**. Lastly, the doublet of peaks between 1150 and 1200 cm^–1^ shows significant differences in general peak shape for the two measured spectra despite similarities in their computed spectra.

The **AzOH-3** compound is easily differentiated from the other derivatives by its triplet between 950 and 1075 cm^–1^. The first peak of this triplet is attributed to CH bending vibrations of the pyrimidine, the peak expected for all compounds described above overlapping with a CO stretch of the methyl (*E*)-3-methoxy acrylate group. The second peak of the triplet is attributed to in-plane CH bending of the pyridine in combination with OH bending at the oxidation site. The last peak of the triplet is attributed to in-plane CH bending of the benzene moiety with the methyl (*E*)-3-methoxy acrylate group, which is also observed and predicted in all other compounds but only observed at the appreciable intensity in the **AzOH-2** and **AzOH-**3 compounds.

### Characterization in Spiked Matrix Samples

**Figure 5:**
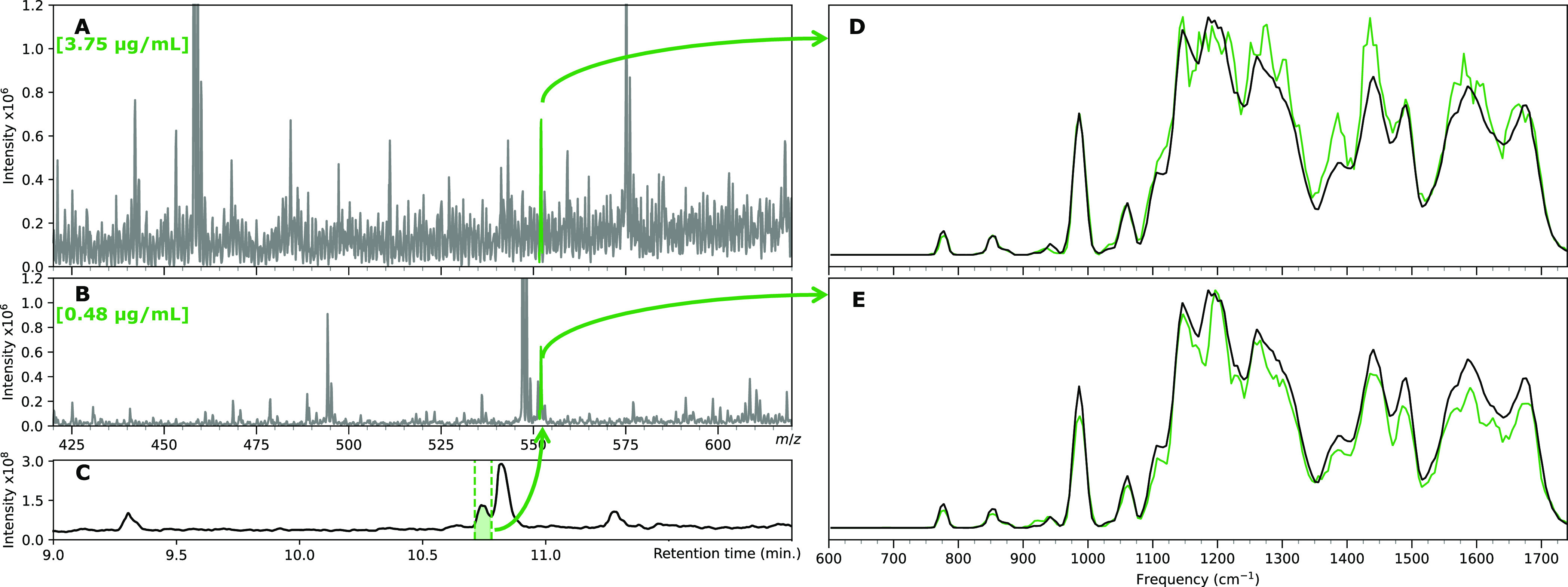
Characterization of the AzOH-2 derivative from the spinach matrix. **A**: Mass spectrum of the spinach matrix spiked with **AzOH-2**; green highlight indicates the ion of interest. **B**: Mass spectrum of the LC-fraction from the spinach matrix indicated in **C** RPLC base-peak chromatogram of the spiked spinach matrix. Fraction collection of **AzOH-2** is indicated by the dashed lines, where switching of the diverting valve occurred. **D**: IRIS spectrum of **AzOH-2** from the spinach matrix (green trace) and IRIS spectrum of the **AzOH-2** reference compound in MeCN (black trace), **E**: IRIS spectrum of **AzOH-2** from fractionated spinach (green trace), and IRIS spectrum of the **AzOH-2** reference compound in MeCN (black trace).

Although the characterization of the agrochemical derivatives in laboratory samples is valuable, we wanted to evaluate the ability of IRIS to characterize the agrochemicals in spiked matrix samples relevant to an agricultural application. Figure 5 depicts two experimental approaches for identifying these compounds from a spinach matrix. In the first approach, the spinach matrix is spiked with approximately 3.75 μg/mL **AzOH-2**, after which it is directly infused into the QIT after spiking with 1 μL of 86 mM CsCl solution to allow the [M + Cs]^+^ adduct at *m*/*z* 552 to be isolated. In this approach, ion suppression from the matrix compounds is the limiting factor in obtaining a clean IRIS spectrum. We know from the measurements described above that the derivatives can be measured dissolved in MeCN at concentrations well below 1 μg/mL. Nevertheless, from Figure 5D, it can be noted that the IRIS spectrum obtained from the matrix matches the spectrum measured of the reference compound in MeCN. However, as a concentration of 3.75 μg/mL is relatively high for regulatory metabolism study samples, a second approach involving fractionating the **AzOH-2** compound from the spinach matrix was pursued. For this, the spinach matrix was spiked with 0.48 μg/mL **AzOH-2,** which was fractioned from the matrix using HPLC. First, the elution time of **AzOH-2** was determined, after which five fractions were collected using a total of 10 μL sample for five fractions, as shown in Figure 5C, yielding a volume of approximately 140 μL, which is diluted approximately 1:1 with MeCN and spiked with 1 μL of 86 mM CsCl solution before infusion into the ESI for IRIS characterization. What can be observed from the mass spectra depicted in Figure 5A, B is that the signal to noise of the *m*/*z* 552 ion is significantly improved in the fractionated analysis. The IR ion spectrum obtained from isolating the 552 *m*/*z* ion is shown in Figure 5E and is nearly identical to the reference spectrum. These results show that IR spectra obtained from matrix samples do not significantly differ from those obtained from laboratory samples of reference standards and suggest that assigning an isomer present in an actual environmental or biological sample would be realistically achievable at these relevant concentrations.

**Figure 6:**
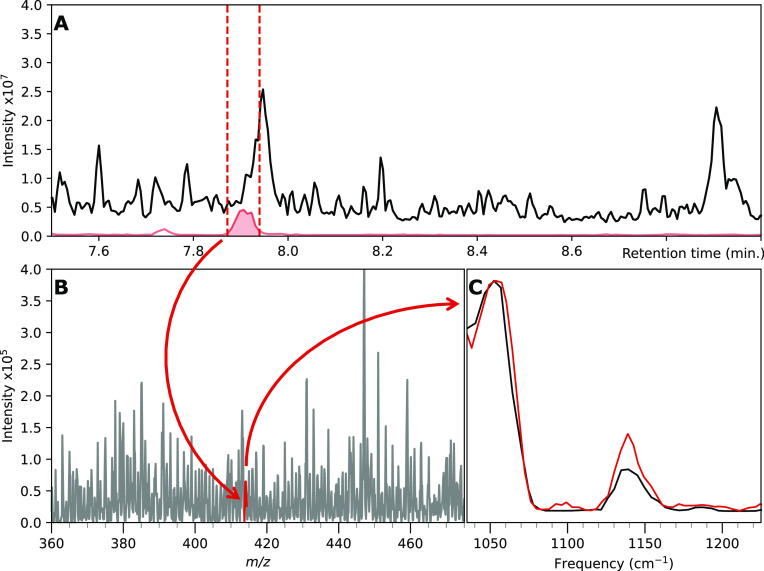
Characterization of the BvOH-1 derivative from the tomato matrix. **A**: RPLC base-peak chromatogram of the spiked tomato matrix run in positive mode (black trace) and base peak chromatogram of the spiked tomato matrix where the QIT was set to isolate at 414 m/z in positive mode. **B**: Mass spectrum of the LC fraction from the tomato matrix. **C**: IRIS spectrum of BvOH-1 from the tomato matrix (red trace) and IRIS spectrum of BvOH-1 obtained from spiking at high concentrations in the tomato matrix (black trace).

The sensitivity of IRIS measurements is determined simply by the sensitivity of the LC–MS in the characterization of the ion of interest. To illustrate that IR ion spectra can be obtained from low concentrations in matrix samples, we spiked **BvOH-1** at nanogram per milliliter concentrations. For this, we spiked **BvOH-1** to a 95 ng/mL concentration in a tomato extract matrix. Figure 6A shows the chromatogram from which **BvOH-1** was fractioned five times, yielding approximately 50 μL, diluted approximately 1:1 with MeCN. Figure 6B contains the mass spectrum measured from this fractioned sample from which the deprotonated adduct at 412 *m*/*z* was isolated. The peak for **BvOH-1** is minor in the MS and even challenging to identify in this mass spectrum; however, IRIS characterization still yields the IR ion spectrum presented in red in Figure 6C, which was measured for the differentiating region between 1025 and 1250 wavenumbers. The IRIS spectrum obtained from the fractionated tomato matrix is nearly identical to the IRIS spectrum recorded from the reference compound (black trace) dissolved at 4 μg/mL in the same tomato matrix. The triplet of peaks, characteristic for **BvOH-1**, is visibly centered at 1040 cm^–1^, also found for the reference spectrum. We observe that the peak shape is slightly less pronounced than the spectrum in Figure 2. However, we attribute this to variation in the power of the FELIX IR-FEL between these measurements and those in Figure 2. Nevertheless, it can be determined that the measurement of the reference standard matches that of the spectrum obtained from the fractioned sample illustrating the effectiveness of IRIS characterization for elucidating agrochemicals from complex matrixes at low concentrations using relatively low volumes of sample.

We demonstrate the utility of IRIS characterization for identifying and differentiating structural isomers of agrochemical derivatives in relevant matrixes. When the agrochemical is present at a high concentration, it can be directly measured from the matrix using IRIS. Furthermore, we have demonstrated that an IRIS spectrum can still be measured at lower concentrations after a simple fractionation of the matrix sample using approximately 10 μL of the sample. For this characterization, approximately 1 ng of the agrochemical is used, which, compared to the microgram amounts of substance required for NMR characterization, is a significant improvement and could allow for improved identification of agrochemical byproducts. In the present study, we have only considered the situation of identifying the structure of a single analyte from its possible structural isomers; however, it is also possible to encounter a mixture of (co-eluting) isomeric analytes. While outside the scope of the present work, we point the interested reader to examples in the literature for which two-color, isomer-selective IRIS methods can potentially be used.^51^

In this study, we used reference standards to facilitate the discussion of the implementation of IRIS for the characterization of agrochemical derivatives. However, we also demonstrated that the computationally predicted spectra at the DFT level are sufficient to differentiate closely related isomers and demonstrated a workflow in which IRIS could facilitate reference-free identification. This computational workflow can also guide the acquisition or synthesis of reference standards, potentially reducing costs associated with synthesizing a plethora of reference standards to assign an unknown compound of interest that may be required in a regulatory metabolism study.

## Supporting Information Available

Supplementary data on the hydroxylated derivatives of the two agrochemicals (PDF)
