# Supplementary material for: Structural Elucidation of Agrochemicals and Related Derivatives Using Infrared Ion Spectroscopy
Source: Environ Sci Technol. 2022 Oct 10;56(22):15563–72. doi: 10.1021/acs.est.2c03210 (PMC9671053; doi:10.1021/acs.est.2c03210)
Supplement: Supplementary file 1 — es2c03210_si_001.pdf [file es2c03210_si_001.pdf]

Structural elucidation of agrochemicals and related derivatives using infrared ion spectroscopy

Matthias J.A. Vink[1], Fred A.M.G. van Geenen[1], Giel Berden[1], Timothy J. C. O'Riordan[2], Peter W.A. Howe[2], Jos Oomens[1], Simon J. Perry[2], Jonathan Martens[1*]*

[1]Radboud University, Institute for Molecules and Materials, FELIX Laboratory, Toernooiveld 7, 6525ED Nijmegen, the Netherlands

[2]Syngenta, Jealott's Hill International Research Centre, RG42 6EY, Bracknell, Berkshire, United Kingdom

* Corresponding author(s)

Jonathan Martens (jonathan.martens@ru.nl) Simon J. Perry(simon.perry@syngenta.com)

This Supporting Information includes 8 pages, 8 figures and 2 tables.

Isomers of hydroxylated metabolites of benzovindiflupyr

Figure 1 of the publication shows the four structures for which the computational prediction of the IR spectra was carried out. However, we note that two stereocenters exist in the molecule. The first stereocenter exists in the orientation of the bicyclo [2.2.1] heptane group of benzovindiflupyr, which can be either pointing up or down. Oxidation on the bicyclo [2.2.1] heptane introduces a second stereo center giving rise to four isomers. It can be determined from Figure SI 1 | Overview of benzovindiflupyr derivatives and their enantiomers that this gives rise to two pairs of mirror images (B1 & C2 and B2 & C1). However, these mirror images can not be resolved by IR spectroscopy. As the main research question focuses on the orientation of the oxidation site, it was not considered relevant to attempt separation of the mirror images. Nevertheless, it should be noted that these isomers may exist.

A1

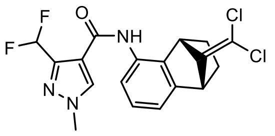

3-Difluoromethyl-1-methyl-1H-pyrazole-4-carboxylic acid ((1SR,4RS)-9-dichloromethylene-1,2,3,4-tetrahydro-1,4-methano-naphthalen-5-yl)-amide

A2

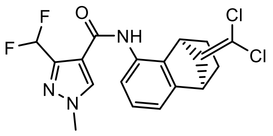

3-Difluoromethyl-1-methyl-1H-pyrazole-4-carboxylic acid ((1RS,4SR)-9-dichloromethylene-1,2,3,4-tetrahydro-1,4-methano-naphthalen-5-yl)-amide

B1

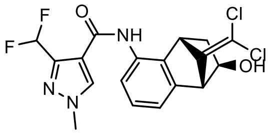

3-Difluoromethyl-1-methyl-1H-pyrazole-4-carboxylic acid ((1SR,2SR,4RS)-9-dichloromethylene-2-hydroxy-1,2,3,4-tetrahydro-1,4-methano-naphthalen-5-yl)-amide

B2

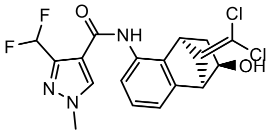

3-Difluoromethyl-1-methyl-1H-pyrazole-4-carboxylic acid ((1RS,2RS,4SR)-9-dichloromethylene-2-hydroxy-1,2,3,4-tetrahydro-1,4-methano-naphthalen-5-yl)-amide

C1

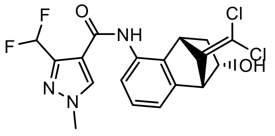

3-Difluoromethyl-1-methyl-1H-pyrazole-4-carboxylic acid ((1S,2R,4R)-9-dichloromethylene-2-hydroxy-1,2,3,4-tetrahydro-1,4-methano-naphthalen-5-yl)-amide

C2

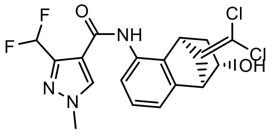

3-Difluoromethyl-1-methyl-1H-pyrazole-4-carboxylic acid ((1R,2S,4S)-9-dichloromethylene-2-hydroxy-1,2,3,4-tetrahydro-1,4-methano-naphthalen-5-yl)-amide

D1

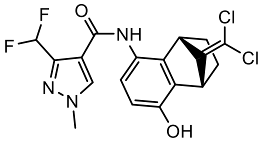

3-Difluoromethyl-1-methyl-1H-pyrazole-4-carboxylic acid ((1R,4S)-9-dichloromethylene-8-hydroxy-1,2,3,4-tetrahydro-1,4-methano-naphthalen-5-yl)-amide

D2

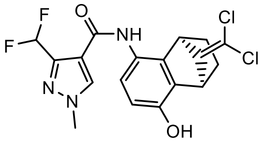

3-Difluoromethyl-1-methyl-1H-pyrazole-4-carboxylic acid ((1S,4R)-9-dichloromethylene-8-hydroxy-1,2,3,4-tetrahydro-1,4-methano-naphthalen-5-yl)-amide

Figure SI 1 | Overview of benzovindiflupyr derivatives and their enantiomers

A1

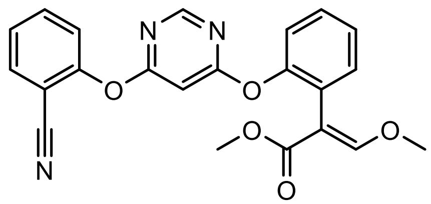

methyl (*E*)-2-(2-((6-(2-cyanophenoxy)pyrimidin-4-yl)oxy)phenyl)-3-methoxyacrylate

B1

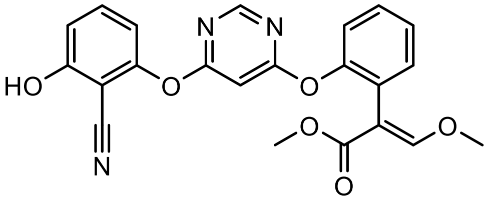

methyl (*E*)-2-(2-((6-(2-cyano-3-hydroxyphenoxy)pyrimidin-4-yl)oxy)phenyl)-3-methoxyacrylate

C1

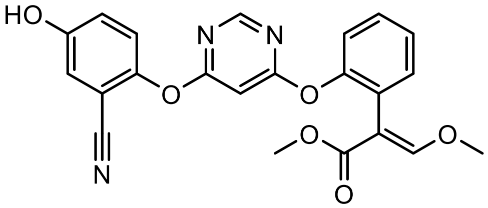

methyl (*E*)-2-(2-((6-(2-cyano-4-hydroxyphenoxy)pyrimidin-4-yl)oxy)phenyl)-3-methoxyacrylate

D1

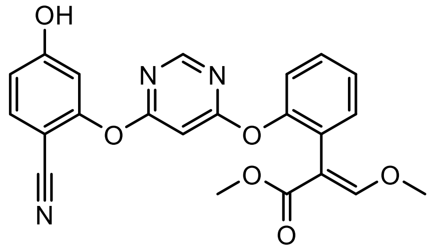

methyl (*E*)-2-(2-((6-(2-cyano-5-hydroxyphenoxy)pyrimidin-4-yl)oxy)phenyl)-3-methoxyacrylate

Figure SI 2 | Overview of azoxystrobin derivatives

Comparison of computed and measured spectra

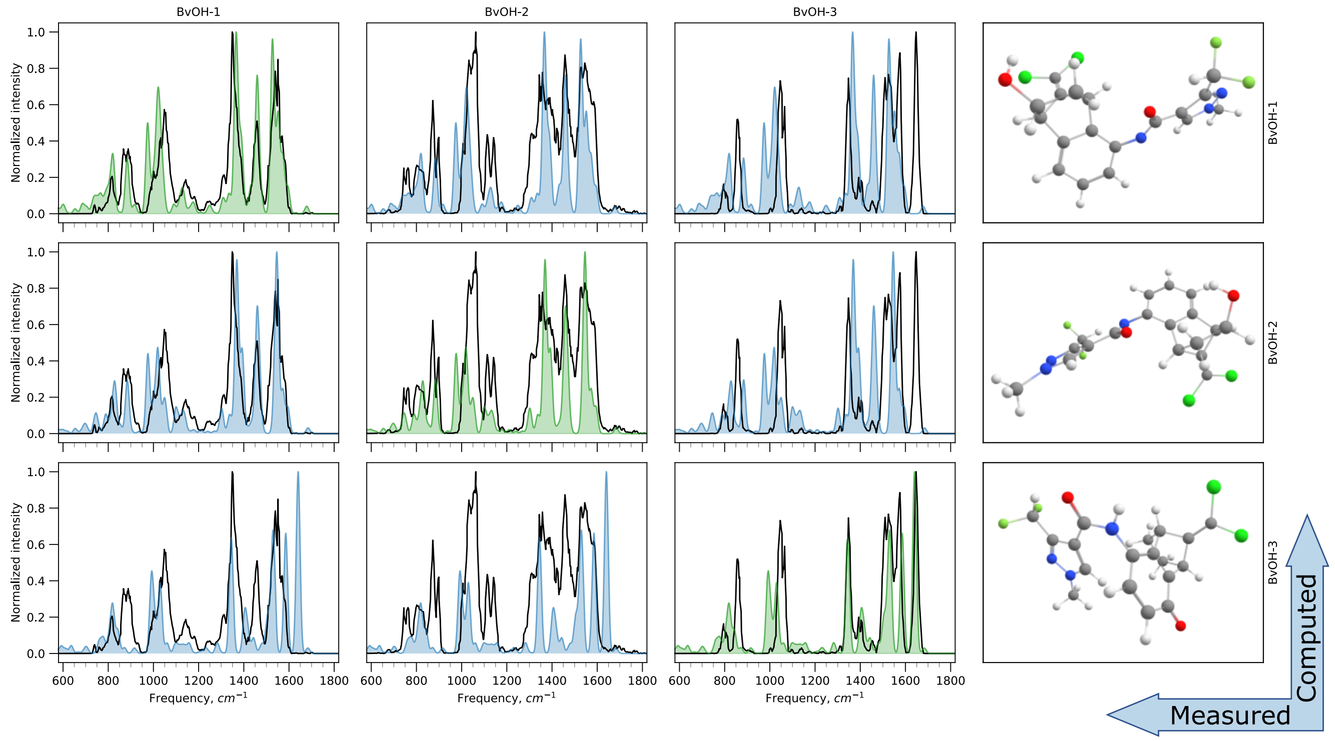

Figure SI 3 | Matrix figure of measured and computed spectra of benzovindiflupyr hydroxylated derivatives. *The measured spectra (from direct infusion of reference standard solutions) are duplicated over multiple rows in the same column, whereas the computed spectra are duplicated over multiple columns in the same row. Additionally, in the last column, the 3D optimized conformation of the matched computed spectrum is provided.*

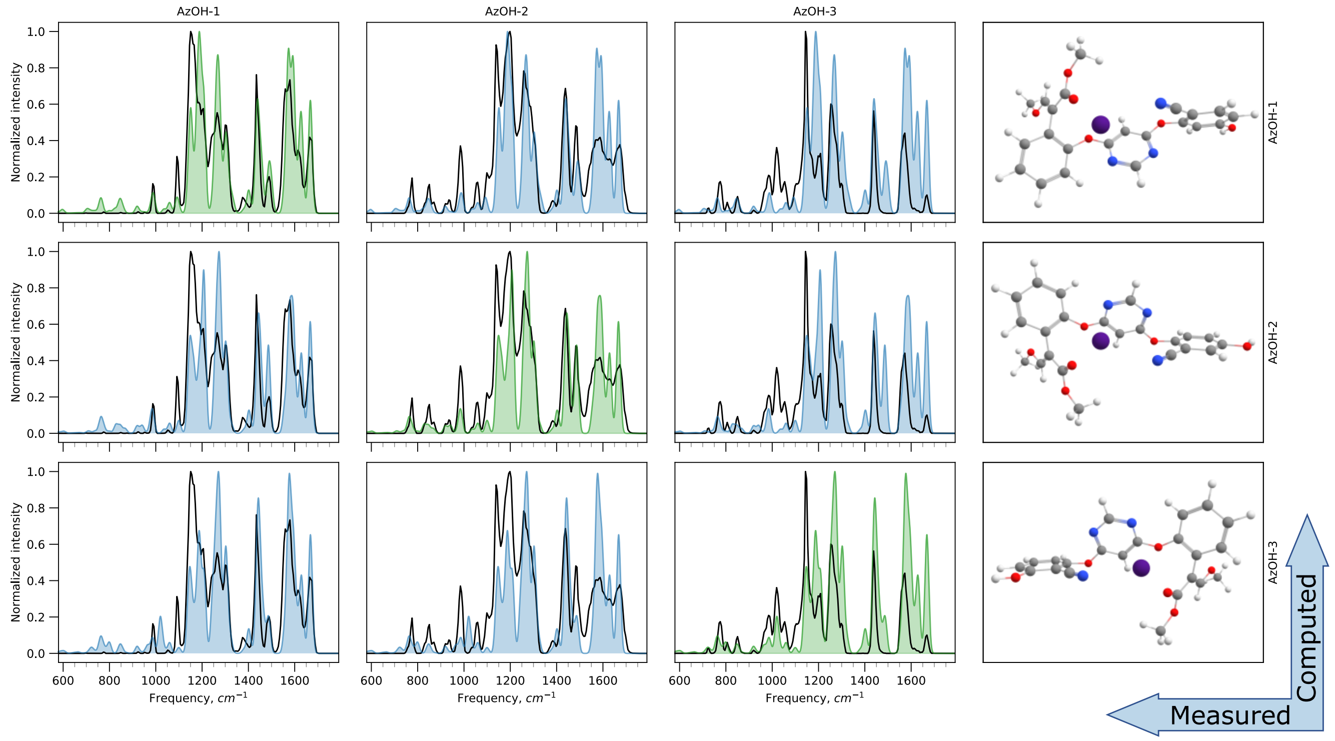

Figure SI 4 | Matrix figure of measured and computed spectra of azoxystrobin hydroxylated derivatives. *The measured spectra (from direct infusion of reference standard solutions) are duplicated over multiple rows in the same column, whereas the computed spectra are duplicated over multiple columns in the same row. Additionally, in the last column, the 3D optimized conformation of the matched computed spectrum is provided.*

Comparison hydroxylated benzovindiflupyr MS/MS spectra

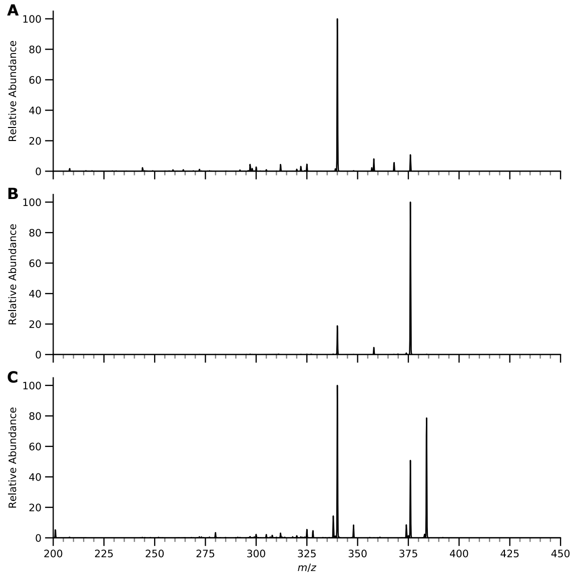

Figure SI 5| Fragmentation spectra of benzovindiflupyr hydroxylated derivatives recorded from direct infusion of the reference standards. ***A****: MS/MS spectrum of deprotonated* ***BvOH-1, B****: MS/MS spectrum of deprotonated* ***BvOH-2, C****: MS/MS spectrum of deprotonated* ***BvOH-3****.*

Comparison hydroxylated azoxystrobin MS/MS spectra

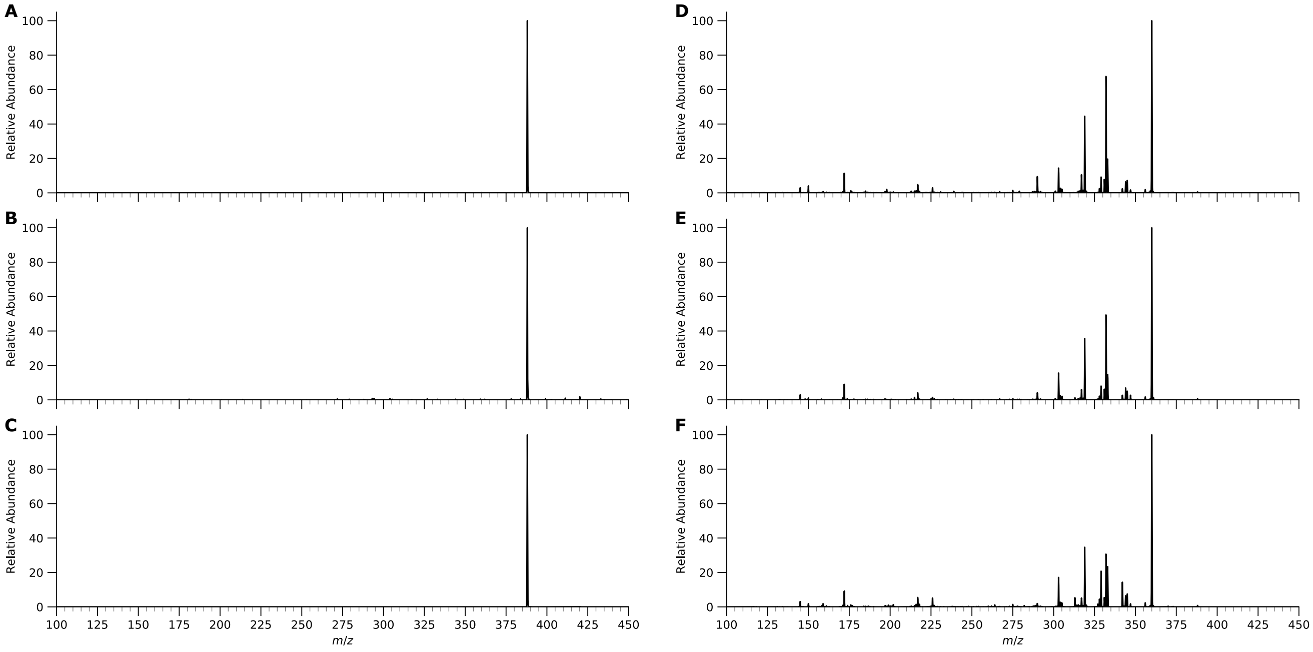

Figure SI 6 | Fragmentation spectra of azoxystrobin hydroxylated derivatives recorded from direct infusion of the reference standards. A: MS/MS spectrum of protonated **AzOH-1**, **B**: MS/MS spectrum of protonated **AzOH-2**, **C**: MS/MS spectrum of protonated **AzOH-3**, **D**: MS^3 spectrum of *m/z* 388 fragment of **AzOH-1**, **E**: MS^3 spectrum of *m/z* 388 fragment of **AzOH-2**, **F**: MS^3 spectrum of *m/z* 388 fragment of **AzOH-3**

Chromatographic separation of the blank matrix samples and comparison with the hydroxylated derivatives

Considering the BPC and EIC of Figure SI 7 and Table SI 1, we can determine that no isobaric compounds elute in the elution range of all hydroxylated derivatives of azoxystrobin in the spinach matrix.

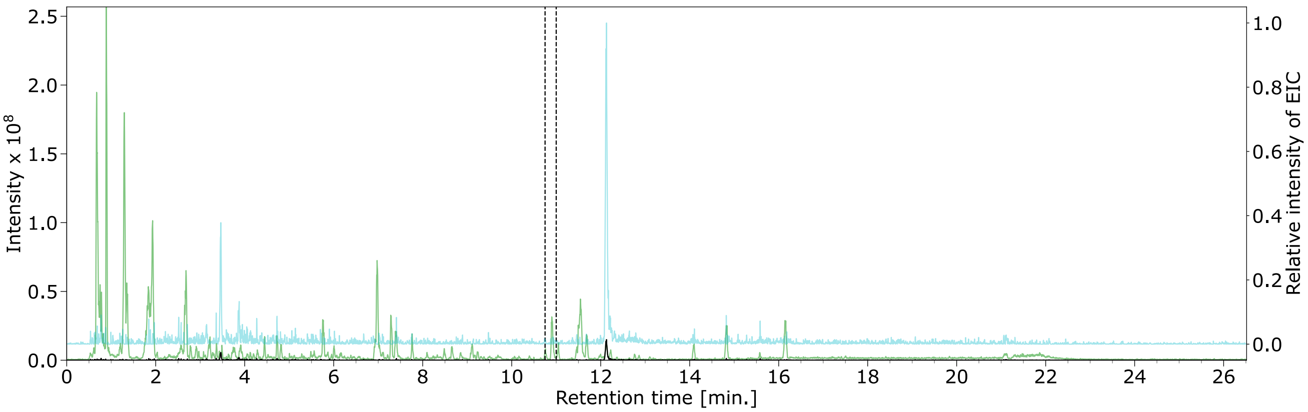

Figure SI 7 | Chromatography analysis of the untreated spinach quality control matrix. The green trace depicts the BPC[+], where the black trace is the EIC of the *m/z* 420 ion which is plotted as normalized intensity as the cyan curve. Black vertical lines indicate the elution range of the azoxystrobin hydroxylated derivatives.

Table SI 1 |Elution times of azoxystrobin hydroxylated derivatives

Name	*Elution time (min.)*
AzOH-1	*11.0*
AzOH-2	*10.75*
AzOH-3	*10.85*

From Figure SI 8 and Table SI 2, we can determine that one isobaric peak is present in the normalized EIC curve. However, upon closer inspection, we can determine that the peak is not close to any of the individual elution times in Table SI 2. Further, we can note that the peak is of very low intensity as it can only be observed in the 'blown out' normalized EIC curve.

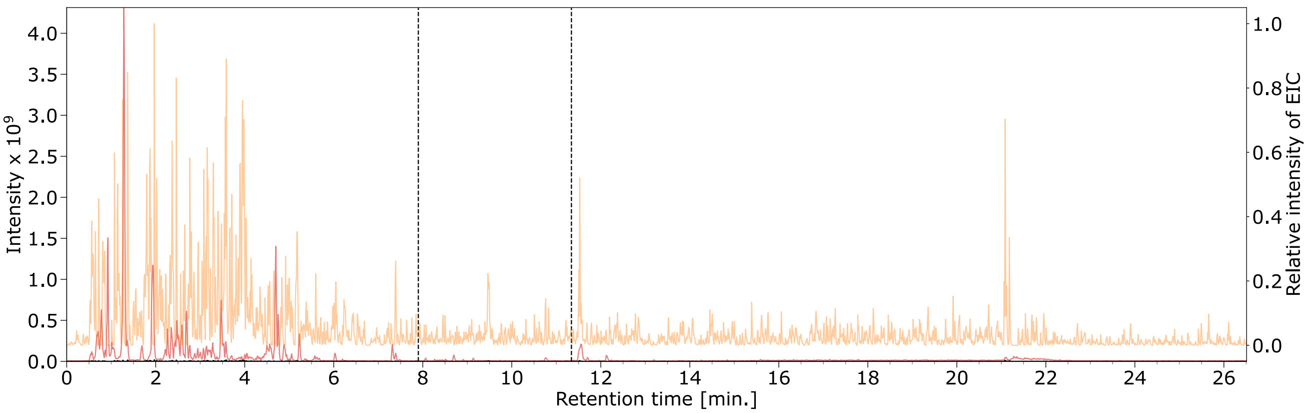

Figure SI 8 | Chromatography analysis of the untreated tomato quality control matrix. The red trace depicts the BPC[-] where the black trace is the EIC of the *m/z* 412 ion which is plotted as normalized intensity as the orange curve.

Table SI 2 |Elution times of benzovindiflupyr hydroxylated derivatives

Name	*Elution time (min.)*
BvOH-1	*7.9*
BvOH-2	*10.88*
BvOH-3	*11.34*
